# Safety and efficacy of panitumumab therapy after metastatic colorectal cancer progression with cetuximab: Experience at a single Japanese institution

**DOI:** 10.3892/ol.2013.1171

**Published:** 2013-02-01

**Authors:** HIROMICHI SONODA, EIJI MEKATA, TOMOHARU SHIMIZU, YOSHIHIRO ENDO, TOHRU TANI

**Affiliations:** Department of Surgery, Shiga University of Medical Science, Shiga 520-2192, Japan

**Keywords:** anti-epidermal growth factor receptor therapy, panitumumab, monoclonal antibodies, colorectal cancer, chemotherapy

## Abstract

Panitumumab (Pmab) is generally considered to be ineffective after the failure of cetuximab (Cmab) therapy in metastatic colorectal cancer (mCRC) patients. However, a few studies have demonstrated that Pmab is an effective treatment for disease progression following Cmab-based regimens in the USA. In the present study, we evaluated the safety and efficacy of Pmab therapy following the failure of Cmab therapy in Japanese patients with mCRC. We performed a retrospective review of the treatment of 16 mCRC patients who tolerated Pmab with clinical benefits after the failure of Cmab therapy between August 2010 and September 2011 at Shiga University of Medical Science. Fourteen of the 16 patients were administered standard Pmab monotherapy (6 mg/kg) intravenously every 2 weeks and the remaining two patients received Pmab with mFOLFOX6 intravenously every 2 weeks. All patients received Pmab chemotherapy until the occurrence of disease progression. Partial radiographic responses (PR) were observed in 2 of the 16 patients and stable disease (SD) was observed in 5 patients. Nine patients had evidence of progressive disease (PD). According to the *KRAS* status, 7 of the 13 (53.8%) patients who had wild-type *KRAS* achieved a high disease control rate (PR + SD). The median progression-free survival (PFS) and overall survival (OS) in the wild-type *KRAS* patients was 96 and 245 days, respectively. Pmab may be an alternative treatment strategy for Japanese patients with mCRC who have experienced failure with standard Cmab-based therapeutic regimens.

## Introduction

Cetuximab (Cmab) is an anti-epidermal growth factor receptor (EGFR) antibody that has been shown to effectively combine with cytotoxic chemotherapy as first-, second- or third-line treatment against wild-type *KRAS* colorectal cancer (1–3). However, it has occasionally been associated with the development of hypersensitivity reactions (HSRs). In patients with severe HSRs, further therapy with Cmab is not possible. Compared with Cmab, HSRs have rarely been observed with panitumumab (Pmab), a fully human IgG anti-EGFR antibody (4). Pmab is considered to be ineffective in patients who experience failure with Cmab therapy, due to evidence that the two antibodies target the same receptor. However, a few studies have indicated that Pmab is effective in patients with refractory metastatic colorectal cancer (mCRC) following the failure of standard therapy, including Cmab-based regimens, in the USA (5,6). However, there are no studies on the efficacy of Pmab therapy after the failure of Cmab therapy in patients from Asian countries. In this study, we aimed to reveal the safety and efficacy of Pmab therapy following disease progression with Cmab therapy in Japanese mCRC patients.

## Patients and methods

### Patient information

We retrospectively reviewed 16 mCRC patients who tolerated Pmab with clinical benefits after the failure of Cmab therapy between August 2010 and September 2011 at Shiga University of Medical Science. Patient medical records were reviewed for previous therapy, toxicity and response assessment. *KRAS* status was retrospectively assessed in patients with readily available tumor tissues. Chemotherapeutic response was assessed using the Response Evaluation Criteria in Solid Tumors (RECIST) (7). The incidence and severity of adverse events (AEs) were measured throughout the study and graded using the National Cancer Institute Common Toxicity Criteria version 4.0 (NCI-CTCAE v4.0).

This study protocol was in accordance with the ethical guidelines established by the Declaration of Helsinki. Written informed consent was obtained from all patients.

### Statistical analysis

Progression-free survival (PFS) and overall survival (OS) were calculated as the interval between the first day of Pmab treatment and the date of proven recurrence or death from any cause, respectively. PFS and OS were estimated using the Kaplan-Meier method.

## Results

### Patient characteristics

The baseline characteristics of the patient population are summarized in Table I. Thirteen patients were male and three were female. The median age was 65 years (range, 53–88 years). All patients had an ECOG (European Clinical Oncology Group) performance status between 0 and 2. The site of the primary tumor was the colon in 5 of the 16 patients (31%) and the rectum in 11 patients (69%). Histologically, the primary tumor was a well-differentiated adenocarcinoma in 6 and a moderately differentiated adenocarcinoma in 10 patients. Ten of the 16 patients (63%) had only one metastatic site (the lungs in 9 cases and liver in 1 case) and 6 of the 16 patients (38%) had two or more metastatic sites, including the liver (5 patients), lungs (5 patients) and lymph nodes (2 patients). Thirteen of the 16 patients (81%) had wild-type *KRAS* and three (19%) had mutant-type *KRAS*. The median number of previous therapies was three (range, 2–6 therapies). Seven of the 16 patients (44%) received four or more previous therapies. All patients received prior irinotecan therapy. Fifteen patients (94%) received prior oxaliplatin and 14 patients (88%) received prior bevacizumab therapy. All patients had been previously treated with Cmab and only two of the 16 patients (13%) discontinued Cmab therapy due to the development of HSRs. Other reasons for stopping Cmab therapy included disease progression (n=13, 81%) and the inconvenience of a bi-weekly schedule (n=1, 6%). Fourteen of the 16 patients (88%) received standard Pmab monotherapy (6 mg/kg) intravenously every 2 weeks, while the remaining two (13%) received Pmab with mFOLFOX6 intravenously every 2 weeks. All patients in this study were administered standard medications (i.e., corticosteroids and antihistamines) prior to Pmab administration in order to prevent HSRs.

### Therapeutic effect

**Figure 1:**
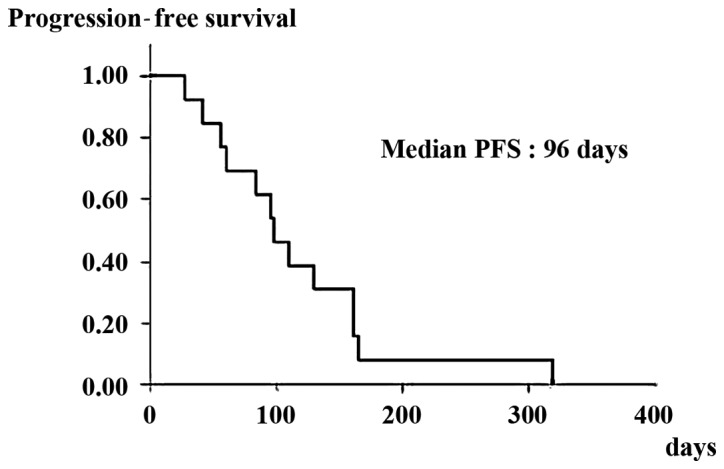
Kaplan-Meier curve demonstrating a median progression-free survival (PFS) of 96 days in patients with wild-type *KRAS*.

**Figure 2:**
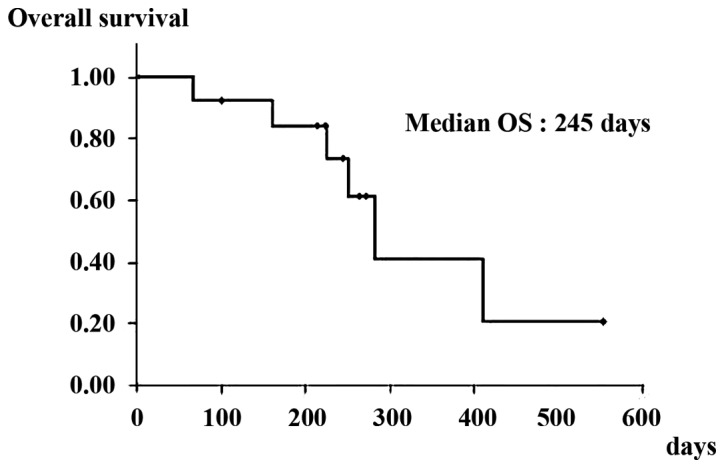
Kaplan-Meier curve demonstrating a median overall survival (OS) of 245 days in patients with wild-type *KRAS*.

All patients received Pmab chemotherapy until disease progression occurred. The median number of Pmab cycles administered was 7 (range, 3–15). Partial radiographic responses (PR) were noted in 2 of the 16 patients (12.5%) and stable disease (SD) in 5 patients (31.3%). Nine patients (56.3%) had evidence of progressive disease (PD). In terms of *KRAS* status, all 3 patients with mutant-type *KRAS* had evidence of PD and 7 of the 13 patients with wild-type *KRAS* (53.8%) achieved a high disease control rate (PR + SD). The median PFS and OS in patients with wild-type *KRAS* was 96 (Fig. 1) and 245 days (Fig. 2), respectively.

### Carcinoembryonic antigen (CEA) levels

One patient did not exhibit a change in CEA level, regardless of tumor status. Four patients achieved a >50% reduction (4934.5 to 793 U/l, 266.4 to 58.3 U/l, 218.2 to 55 U/l and 31.8 to 14.5 U/l), 2 patients had a 25% reduction (459.5 to 296.5 U/l and 34.7 to 22.7 U/l) and 1 patient had a minor reduction (442.8 to 380 U/l) in CEA levels and 8 patients had increased CEA levels. Therefore, approximately half of the patients achieved a reduction in CEA levels.

### Typical case

**Figure 3:**
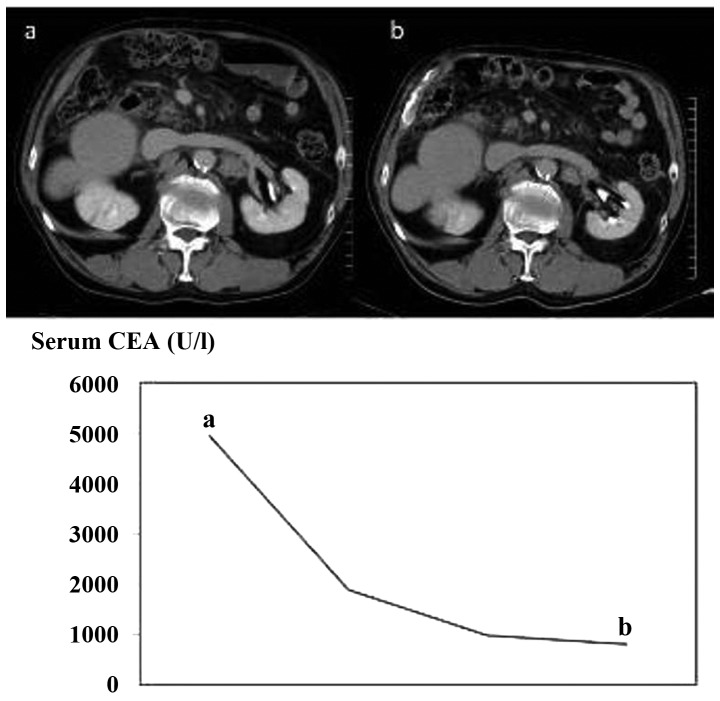
Patient with unique sensitivity to Pmab after various therapies, including cetuximab (Cmab). (a) Before Pmab treatment; (b) 10 months after Pmab treatment. CEA, carcinoembryonic antigen.

A 73-year-old male had been diagnosed with sigmoid colon cancer (T3N1M0 Stage IIIa) 7 years previously, for which he underwent a sigmoidectomy with regional lymph node excision. Two years after sigmoidectomy, the patient underwent partial hepatectomy for solitary liver metastasis. The patient was once again diagnosed with multiple liver metastases several months after the partial hepatectomy, for which hepatic arterial infusion chemotherapy (HAI) was administered. However, due to disease progression despite the HAI, mFOLFOX6, FOLFIRI and Cmab chemotherapy was administered. Thereafter, the patient was also diagnosed with lung and intra-abdominal lymph node metastases and received Pmab therapy, following which disease stability was achieved for ∼10 months and CEA levels decreased from 4934 to 793 U/l (Fig. 3).

### AEs

All patients tolerated Pmab well, with no cases of HSR. Grade 3/4 toxicities included hypomagnesemia in 1 patient and hypocalcemia in another. Other AEs observed included grade 1–2 skin rash in 3 patients, grade 2 hypomagnesemia in 2 patients, grade 1–2 fatigue in 3 patients and grade 1–2 appetite loss in 3 patients. However, none of the patients required discontinuation of Pmab therapy as a result of these AEs.

## Discussion

For ∼40 years, fluoropyrimidine 5-fluorouracil was the only drug available for the treatment of mCRC. However, over the past 10 years, the therapeutic armamentarium for mCRC has expanded significantly with the development and approval of three cytotoxic agents, irinotecan, oxaliplatin and the oral fluoropyrimidine capecitabine (8). During this time, three new biological agents were developed, including the anti-vascular endothelial growth factor (VEGF) antibody bevacizumab and anti-EGFR antibodies Cmab and Pmab. Incorporation of these biological agents into various cytotoxic chemotherapy regimens has transformed mCRC into a chronic illness, where the median OS is currently 24–28 months (9).

Cmab and Pmab bind with a high affinity to EGFRs and prevent the binding of natural growth factor ligands to these receptors (10). This inhibitory effect leads to the repression of subsequent downstream signaling pathways, which mediate cell growth and proliferation, survival mechanisms against chemotherapy and/or radiation therapy, invasion/metastasis and even angiogenesis. Cmab or Pmab therapy is not considered to be effective following disease progression with Pmab or Cmab therapy, respectively. However, Cmab is a chimeric antibody that is associated with the development of HSRs. The development of severe HSRs means premature termination of drug therapy is necessary (11,12). Pmab is a fully human IgG2 antibody and, in contrast to Cmab, infusion-associated reactions are usually minor in severity and grade 3/4 reactions are rarely experienced. As a result, premedication is not usually recommended when Pmab therapy is administered (4). In our study, Pmab was effective even after the failure of Cmab. In Western countries, there have been a number of previous studies on the safety and clinical efficacy of Pmab following disease progression with Cmab therapy (5,6). In the present study, >60% of patients who experienced disease progression with Cmab therapy achieved varying levels of clinical benefit, including disease control and a reduction in levels of the serum tumor marker CEA, with Pmab therapy. Our results are similar to those reported by Power *et al* on their clinical experience at the Memorial Sloan-Kettering Cancer Center (6). Notably, we were able to prolong PFS by ∼3 months and the OS by ∼8 months by administering Pmab, even after third-line chemotherapy.

At present, it is not entirely clear why patients who had disease progression on Cmab were able to derive clinical benefits from Pmab. Recently, Montagut *et al*(13) revealed that the presence of the acquired EGFR ectodomain mutation (S492R) may provide a molecular explanation for the clinical benefits of Pmab therapy in a subset of patients with mCRC who did not respond to treatment with Cmab. Another possibility is that the two antibodies may inhibit EGFR signaling via separate mechanisms. To explore these possibilities, Freeman *et al*(14) performed epitope mapping of the two antibodies and revealed that Pmab and Cmab bind to the same surface-exposed amino acids in domain III of EGFRs and this inhibited the binding of all known EGFR ligands. However, formal X-ray crystallographic studies showed that the humanized anti-EGFR antibody matuzumab interacts with an epitope on EGFRs that is distinct from the ligand-binding region on domain III and the Cmab epitope (15,16). Matuzumab indirectly blocked ligand-induced receptor activation by sterically preventing the domain rearrangement and local conformational changes that must occur for high-affinity ligand binding and receptor dimerization. Structural studies of a different humanized anti-EGFR antibody, nimotuzumab, revealed a novel mechanism in which nimotuzumab blocked EGF binding while allowing the EGFR to adopt its active conformation. By interfering with only ligand-dependent EGFR activation, nimotuzumab was able to reduce EGFR signaling to a basal, ligand-independent level. Taken together, these studies suggest that the various anti-EGFR antibodies may inhibit EGFR-mediated signaling through different mechanisms. As a result, it is also conceivable that distinct mechanisms of resistance may develop to the respective anti-EGFR antibodies.

In conclusion, Pmab therapy may represent an alternative treatment strategy for patients, Japanese or otherwise, with refractory mCRC who have experienced failure with standard therapies, including Cmab-based regimens. Our relatively small clinical experience suggests that Cmab and Pmab may exert their antitumor activity via different mechanisms, however, further study is required to investigate this hypothesis.

## Figures and Tables

**Table I:** Baseline and clinical characteristics of the metastatic colorectal cancer patients.

Characteristics	Value
Total number (%)	16 (100)
Male/female (%)	13/3 (81/19)
Age (years)	
Median	65
Range	53–88
Performance status (%)	
0	7 (44)
1	5 (31)
2	4 (25)
Primary tumor site (%)	
Colon	5 (31.25)
Rectum	11 (68.75)
Histology (%)	
Well-differentiated	6 (37.5)
Moderately differentiated	10 (62.5)
Poorly differentiated and others	0 (0)
Number of metastatic sites (%)	
1	10 (62)
2	3 (19)
≥3	3 (19)
Sites of metastases (%)	
Liver	6 (38)
Lungs	14 (88)
Lymph nodes	2 (13)
Other	5 (31)
*KRAS* status (%)	
Wild-type	13 (81)
Mutant	3 (19)
Prior therapeutic regimens (%)	
2	6 (38)
3	3 (19)
≥4	7 (44)
Prior oxaliplatin therapy (%)	15 (94)
Prior irinotecan therapy (%)	16 (100)
Prior bevacizumab therapy (%)	14 (88)
